# Electric parameter prediction of rod pumping units based on N-HiTS model

**DOI:** 10.1371/journal.pone.0326973

**Published:** 2025-07-03

**Authors:** Xiangyu Li, Zhiqing Liu, Chunhua Yuan, Zhupei Liao

**Affiliations:** School of Automation and Electrical Engineering, Shenyang Ligong University, Shenyang, Liaoning Province, China; GH Raisoni College of Engineering and Management Pune, INDIA

## Abstract

The prediction of the operation state of rod pumping unit is one of the important issues in rod pumping engineering. The electrical parameters of the driving motor are gradually becoming the main research direction because of its stability and long-term acquisition. However, there are problems with the existing collection of electrical parameter data, such as a large volume of highly repetitive data within a day, the operation conditions remaining the same for a long time, and fault conditions being rare. This article proposes a new method of combining electrical parameter sequences to predict the operating conditions of rod pumping units. Firstly, an improved mechanism model was established from the rod pumping unit to the driving motor, generating electrical parameter data for typical fault conditions and composite conditions. Secondly, by combining the generated electrical parameter sequences into a time series, the Neural Hierarchical Interpolation for Time Series (N-HiTS) model from the NeuralForecast library is used for prediction. The experimental results show that the proposed method can predict real electrical parameters, generate electrical parameters, and simulate electrical parameters under composite fault conditions. The approach of generating electrical parameters and making predictions can effectively address the issue of insufficient electrical parameter samples, thus enabling a more precise prediction of the operating state of the pumping unit, and adjusting production activities of oil wells.

## 1 Introduction

Rod pumping is a widely used oil recovery method in the modern oil production industry [1,2]. Especially in China, about 80 percent of the oil fields are operated by rod pumping [3]. Usually, pumping units need to be kept running for a long time. When the pumping unit breaks down, the maintenance and time cost are high, and the economic loss is easy to be caused. Therefore, it is very significant to monitor the operating condition of the pumping unit [4].

Downhole conditions are often complex, and the fluid extracted contains gravel, wax, and corrosive substances. The long working time of the pumping unit will cause the loss of related components such as rod and plunger. Therefore, it is necessary to reserve the relevant parts for replacement when the loss is serious. By way of prediction, it can not only help to prepare replacement parts in time, but also avoid the waste of resources caused by hoarding too much material. Moreover, the oil production situation of the oilfield can be judged by observing the forecast data, so as to optimize the production management of the oilfield [5].

Dynamometer cards (DCs) are the most commonly used data to reflect downhole conditions and pumping unit operating conditions [6,7]. DCs are usually obtained through sensors mounted on the pumping unit [8]. However, sensors are mostly battery-powered and difficult to work for a long time. And the installation and replacement need to suspend the work of the pumping unit, which loses some production benefits. Therefore, for observing and analyzing the operating condition of the rod pumping unit more conveniently, more and more attention has been paid to the method of taking the electric parameters as the research object [9–11].

Compared with dynamometer card (DC) datas, the acquisition of electric parameters is more convenient and stable. Electrical parameters can be collected in the distribution box that drives the motor. The motor is connected with the reducer box through the belt. When the motor is running, the high-speed operation of the motor converts the vertical motion of the smooth slide rod under the action of the reducer box and the four-link structure, so that the pumping unit can work normally [12]. There is a strong coupling exists between motor operation and variations in downhole conditions. For one thing, when the motor runs abnormally, it will affect the normal oil production process of the rod pumping unit; for another, when the downhole conditions change, the rod movement is abnormal, which makes the motor speed fluctuate. Therefore, electric parameters can effectively indicate the operating condition of the rod pumping unit. Sensors that collect electric parameters are installed in the distribution box, and the data can also be accessed via mobile devices. Compared to traditional DC acquisition, collecting electric parameters is more cost-effective and convenient. The electric parameters can be collected stably over a long period of time, so the electric parameters are more helpful for predicting the state of the rod pumping unit.

Continuous operation of the pumping unit causes sensors to generate large volumes of electrical data, placing a heavy burden on the host computer’s memory. Feeding these raw parameters directly into a model also dramatically increases computational load. To mitigate both issues, we propose a electric-parameter fusion approach that preprocesses the model inputs. Electrical parameters are sampled at fixed intervals and concatenated in temporal order, a strategy that cuts redundancy yet preserves diverse electrical information across multiple days and convenient for later data prediction.

Some methods have been studied in the existing reports for the establishment of prediction models in rod pumping systems. For example, temporal neural network [13], an innovative time-series analytics approach [14], etc. The methodology used in this paper is from the N-HiTS prediction model [15] in the NeuralForecast library. NeuralForecast offers a number of neural network models for forecasting, including Neural Basis Expansion analysis for interpretable Time Series (N-BEATS) [16], Recurrent Neural Network (RNN) [17], and Long Short-Term Memory (LSTM) [18]. N-HiTS model is proposed on the basis of N-BSATS and has achieved good performance in several classical time series data sets, such as Electricity Transformer Temperature, San Francisco Bay Area Highway Traffic and Weather datasets [19–21]. For the modern oil production industry site, there are basically many pumping units working together. Therefore, so as to obtain the working status of each pumping unit equipment, the motor data that needs to be collected will be very large. In addition, in most cases, the composition of the motor data may vary depending on the motor model or the model of the pumping unit. The N-HiTS does not require model inputs to have specific formats and shapes, which can better adapt to different types of time series. Compared with the traditional prediction models LSTM and RNN, N-HiTS not only improves the prediction accuracy, but also significantly reduces the prediction time and calculation consumption of the model. N-HiTS is therefore able to process abundant different types of motor data.

Based on the inspiration of the above research, this paper uses the mechanism model to generate the motor power data, and uses the N-HiTS model to predict the electrical parameter sequence. The main contributions are as follows: Firstly, based on the relationship between the operating state of the motor and the rotation of the crankshaft, an electrical parameter generation model from the rod pumping unit to the driving motor was established. Generate electric parameters based on changes in suspension load as input. Secondly, based on existing research, the electrical parameter generation model was optimized. On the one hand, the input part of the model is equipped with an automatic input format adjustment function. This function can automatically recognize and adjust the time sequence of changes in suspension load. On the other hand, the model can simulate the equilibrium state of the pumping unit. And it can be combined with a single fault to generate working condition data of composite fault types. Finally, the generated electrical parameter sequences are combined into different time series, and the electrical parameter data is measured by down sampling to solve the redundancy problem of the electrical parameter dataset. Use the prediction model N-HiTS to predict the electrical parameter time series and compare it with other prediction models. The feasibility of generating electrical parameter sequences for predicting the operating status of rod pumping units has been verified, which can effectively alleviate the problem of insufficient electrical parameter samples.

## 2 Methods

### 2.1 Electric parameter generation model

**Fig 1:**
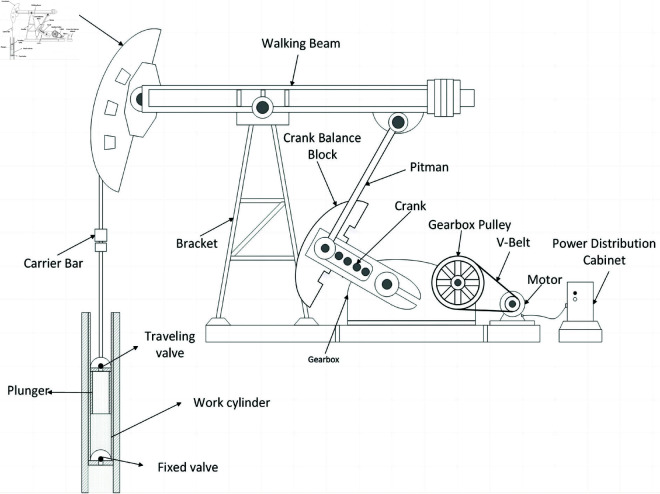
Motor driven rod pumping unit construction.

The structure of rod pumping unit driven by motor is shown in Fig 1. The drive motor is connected to the pulley on the gearbox by a belt. When the motor is running at high speed, the belt drives the gearbox pulley to rotate, and then drives the crank shaft on the gearbox to rotate. The crank connecting rod mechanism is used in the pumping unit to convert the rotating motion generated by the crank shaft into the up and down swing of the floating beam. Therefore, the horsehead can drive the sucker rod to move up and down to draw the oil to the surface to meet the production demand.

Downhole conditions are complex, resulting in irregular and unstable load fluctuations in rod pumping units. Variations in these conditions lead to changes in the suspended point load, which in turn cause fluctuations in motor power, speed, and current. According to the change of suspension load, the electric parameter generation model generates the motor power through the relationship between motor rotation and crank rotation [22].

Therefore, on the basis of the coupling relationship between the pumping unit and the motor, the power of the motor will also show a similar change. For rod pumping units, the relationship between the motor power and the net torque of the crank shaft on the reduction box is:

$$\left\{ \begin{array}{c} P_{motor}=\frac{n_{m}M_{cr}}{i\eta 9549}\\ i=i_{1}\frac{D}{d}\\ \eta =\eta_{1}\eta_{2} \end{array}\right.$$ (1)

where *P*_*motor*_ denotes the motor power (KW); *n*_*m*_ denotes the motor rotational speed (rpm); *M*_*cr*_ denotes the net torque transmitted from the motor to the crankshaft (KN·m); *i* is the transmission ratio from the motor to the gearbox; *i*_1_ is the transmission ratio of the gearbox; *D* and *d* represent the diameters of the gearbox pulley and motor pulley, respectively (mm); η denotes the overall transmission efficiency; $\eta_{1}$ and $\eta_{2}$ represent the belt transmission efficiency and gearbox transmission efficiency, respectively.

**Fig 2:**
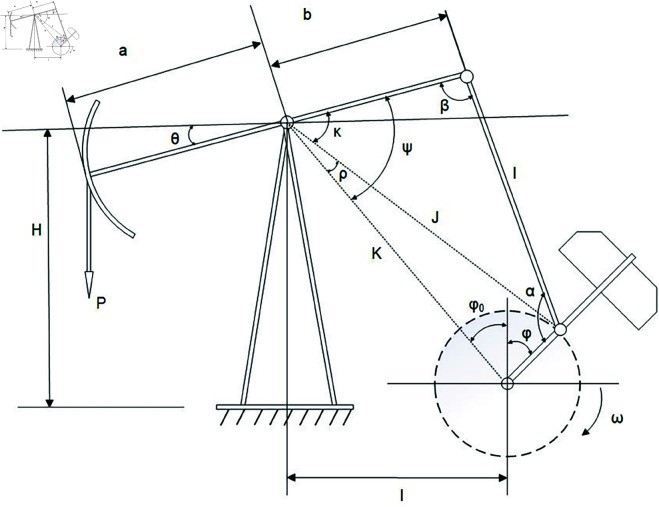
Physical structure of rod pumping unit.

For the crank balanced rod pumping unit, its physical structure is shown in Fig 2. The *M*_*cr*_ is the difference between the torque *M*_*p*_ caused by the suspension load and the torque *M*_*c*_ acted by the counterweight of the crank (KN·m), as follows:

$$M_{cr}=M_{p}-M_{c}$$ (2)

The torque *M*_*p*_ caused by the suspension load is also known as the well load torque and is defined as:

$$\left\{ \begin{array}{c} M_{p}=\bar{TF}\cdot (P-B)\\ \bar{TF}=\frac{a\sin\alpha r_{crank}}{b\sin\beta } \end{array}\right.$$ (3)

where *B* denotes the structural unbalance value (KN); *a* and *b* is the lengths of the forearm and back arm of the walking beam, respectively (m); α is the angle formed by the crank and connecting rod, in degrees(∘); *r*_*crank*_ is the crank radius, m; β is the angle between the back arm of the beam and the connecting rod, in degrees(∘). α and β are functions of crank angle φ(∘). For certain types of pumping units, the torque factor at each stroke can be obtained according to different crank angles. Therefore, α and β are calculated as follows:

$$\left\{ \begin{array}{c} \beta =\frac{\arccos b^{2}+l^{2}-K^{2}-r^{2}+2\;Kr\cos\left(\varphi +\varphi_{0}\right)}{2bl}\\ \varphi_{0}=\arctan\left(\frac{I}{H-G}\right)\\ \alpha =360^{\circ }-\beta -\psi -\varphi -\varphi_{0}\\ \psi =\kappa +\rho \\ \kappa =\arcsin(\frac{l\sin\beta }{J})\\ J=\sqrt{l^{2}+b^{2}-2lb\cos\beta }\\ \rho =\arcsin\left(\frac{r\sin\varphi +\varphi_{0}}{J}\right) \end{array}\right.$$ (4)

where *l* denotes the effective length of the connecting rod (m); *K* is the distance from the center of the crankshaft to the center of the walking beam (m); *I* is the horizontal distance from the center of the beam shaft to the center of the crankshaft (m); *H* is the vertical distance from the center of the beam shaft to the base (m); The φ is the angle of clockwise rotation of the crank from the 12 o’clock position. The value of φ can be obtained by the ratio of the speed of the motor to the total transmission ratio, as follows:

$$\left\{ \begin{array}{c} \omega =\frac{n_{m}}{i}\\ \varphi =\varphi_{0}+\sum_{n=0}\omega_{n}t_{interval} \end{array}\right.$$ (5)

where ω is the rotational speed of the crank (rpm); $\varphi_{0}$ is the initial angle of the crank, in degrees(∘), and $t_{interval}$ stands for interval at which the crank speed changes. The value of *M*_*c*_ is defined as follows:

$$\left\{ \begin{array}{c} M_{c}=M_{cmax}\sin\varphi \\ M_{cmax}=W_{cb}R+W_{c}R_{c} \end{array}\right.$$ (6)

Where *W*_*cb*_ is the total weight of all counterweights mounted on the crankshaft (KN); *W*_*c*_ is the weight of the crank itself (KN); *R* is the distance from the crankshaft to the center of gravity of the counterweights (m); *R*_*c*_ is the distance from the crankshaft to the center of gravity of the crank (m). Therefore, for a crank balanced pumping unit, the net crank torque can also be expressed as:

$$M_{cr}=\bar{TF}(P-B)-M_{cmax}\sin\varphi$$ (7)

**Fig 3:**
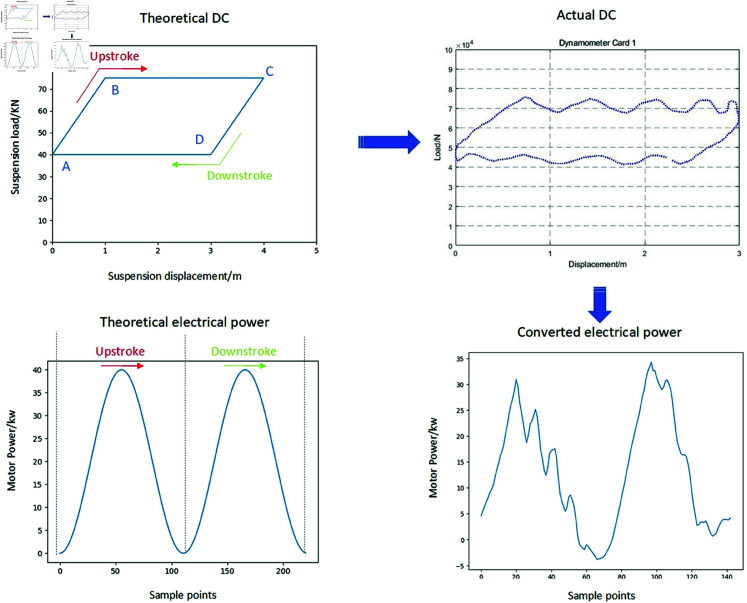
Schematic diagram of converting DCs into motor power curve.

Through Eqs 1 to 7, a mechanistic model is established to map the polished rod load to the motor power. Since the DC provides a clear representation of the relationship between the polished rod load and displacement, it becomes feasible to synthesize electric parameter samples from existing DCs data, as illustrated in Fig 3. Moreover, both the DC and electric parameter exhibit intrinsic periodicity. To ensure consistent alignment of input sequences for subsequent model training and forecasting, a fixed starting point is specified within each cycle.

The DC signal collected by field sensors reflects the load–displacement trajectory within a single stroke of the sucker rod, which comprises an upstroke and a downstroke. Under normal operation, the displacement ranges for both strokes are expected to be equal. However, due to the harsh operating conditions in oilfields, sensor malfunction is common. To address such issues, any discontinuities in the DC data must be preprocessed before being input into the model. In the example shown, the discontinuity consists of only a single missing data point, which can be effectively reconstructed through manual interpolation. As shown in Fig 3, the upstroke begins at the bottom dead center (point A), with load increasing from point A to point B. During this phase, both the traveling and standing valves remain closed. Once point B is reached, the standing valve opens, and fluid is drawn into the pump barrel as the rod travels to point C, marking the top dead center and the end of the upstroke. The downstroke then proceeds from point C to point A. At point C, where the standing valve is again closed. At point D, the traveling valve opens, and the plunger discharges fluid into the tubing until the rod returns to point A, completing one full pumping cycle.

**Fig 4:**
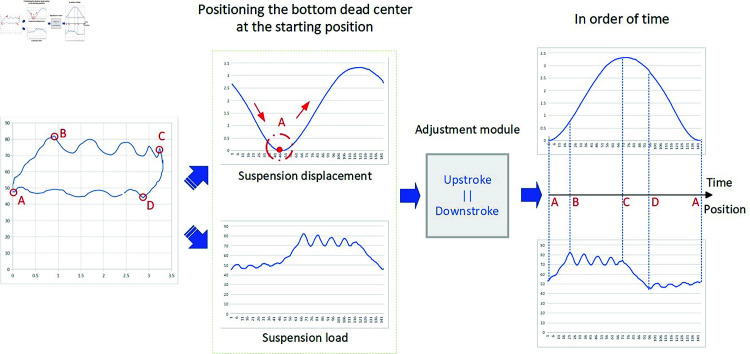
Automatic adjustment of input data by electrical reference production model.

Due to variations in pumping unit models across different oilfields, the specifications of DC sensors also vary. In general, the DC sensors typically collect 144 or 250 samples per stroke cycle, including both polished rod displacement and load. As a result, DC data cannot be directly used as input for generating electrical parameters. To resolve this, in our MATLAB preprocessing routine, the sampling point corresponding to the minimum displacement—i.e., the bottom dead center (point A)—is identified and set as the starting point of the cycle. The time-series data is then reordered accordingly to ensure correct temporal alignment before being input into the mechanistic model. For instance, with 250 sampling points, if point A is detected at index 46, the reordered sequence becomes: samples 46 to 250, followed by samples 1 to 45. The electric power profile of the pumping unit during this operating cycle can be obtained through transformation using the mechanism model, as shown in Fig 4.

At present, the collection of electric parameters is not widely used in most domestic oil fields, and the data source for diagnosing the fault of pumping unit is still DC. The rod pumping unit needs long-term stable operation to meet the industrial production benefits, and the structural design of the rod pumping unit determines the low failure rate. Therefore, it is difficult to obtain multiple types of electrical parameters that reflect typical fault states in a short period of time. The above mechanism model can generate corresponding electrical parameters based on the DCs from different oil fields, solving the problem of insufficient electrical parameter samples in fault states. This model can automatically adapt to different acquisition formats of indicator diagrams and has stronger applicability.

### 2.2 N-HiTS forecast model

**Fig 5:**
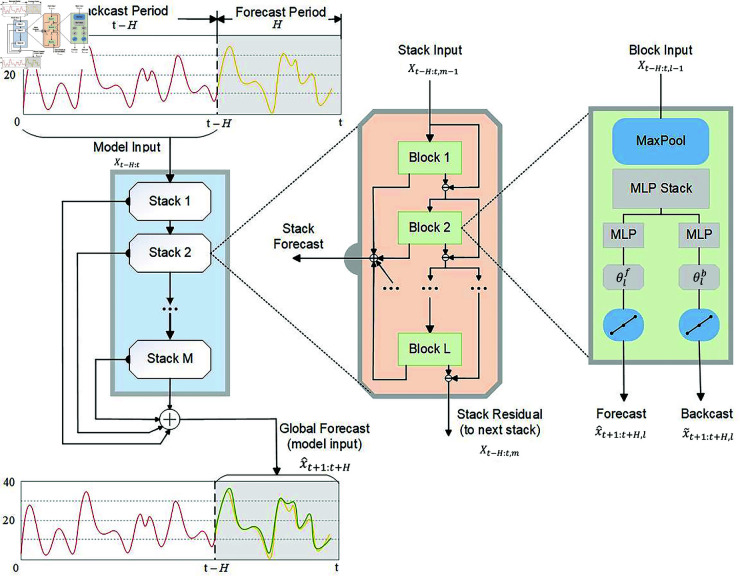
Structural diagram of N-HiTS prediction model.

Structurally, the N-HiTS model is sequentially connected with *M* stacks, each containing *L* blocks. Within each stack, the connections between blocks are made using a hierarchical doubly residual topology structure. The principle and structure of N-HiTS are shown in Fig 5.

**Fig 6:**
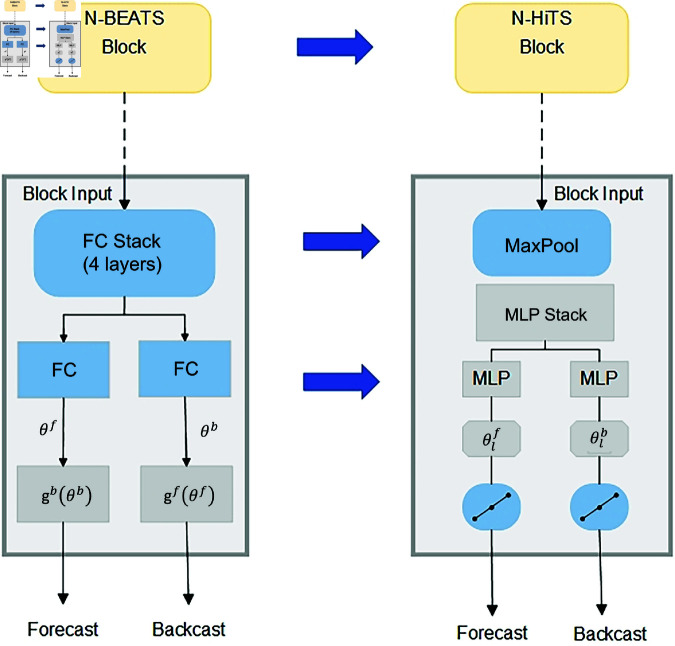
Contrast of N-HiTS and N-BEATS blocks.

The N-HiTS model is an improvement on the N-BEATS model. Compared with the N-BEATS model, N-HiTS has improved the structure and function of the block, optimized the processing capacity of the input signal, and improved the computing capacity of the model, as shown in Fig 6. In the structure of N-BEATS model, the block mainly adopts the fully connected layer, and the time series is predicted forward and backward respectively. On this basis, N-HiTS proposes to replace the fully connected layer structure with MLP, and adds MaxPool layer to the input of the block to improve the processing capacity of the input sequence. For a given time series, the initial input to the network is *x*_*t*−*H*:*t*_, *H* is the length predicted by the model. Each stack contains *L* blocks, so multiple stacks in the network can use different basis functions to learn different features of the input data. Therefore, each stack will output a different prediction, called a local prediction. The final prediction result of the network is a combination of the forecast results of each stack, called the global prediction.

#### 2.2.1 Multi-rate signal processing.

A MaxPool layer of size *k*_*l*_ is set at the input of each block, where *l* refers to the *l*-th block. The input *x*_*t*−*H*:*t*_ of the maximum pooling layer, with a length from *t*–*H* to *t*. Before the effective input signal enters the MLP, the MaxPool layer can cut the high-frequency or small-time signals in the input signal, making the MLP more efficient in processing the large-time or low-frequency parts of the signal. In addition, Maxpools with larger *k*_*l*_ show better cutting results. This operation is called multi-rate signal sampling. The expression for multi-rate signal sampling is as follows:

$$x_{t-H:t,l}^{\left(p\right)}=MaxPool(x_{t-H:t},k_{l})$$ (8)

where *p* represents forward or backward.

#### 2.2.2 Non-linear regression.

The input signal is subsampled at the MaxPool layer and then fed into the MLP. MLP uses nonlinear aggregation of input signals to learn the hidden layer vector $h_{l}\in {\mathbb{R}}^{n\times h}$. After linear projection, the forward coefficients $\theta_{l}^{f}$ and backward coefficients $\theta_{l}^{b}$ are output respectively, as follows:

$$\left\{ \begin{array}{c} h_{l}=MLP_{l}(x_{t-H:t,l}^{\left(p\right)})\\ \theta_{l}^{f}=LINEAR^{f}(h_{l})\\ \theta_{l}^{b}=LINEAR^{b}(h_{l}) \end{array}\right.$$ (9)

#### 2.2.3 Temporal interpolation.

Long-term time series forecasting remains a significant challenge due to the increasing complexity of temporal dependencies over extended horizons. For most prediction models, the length *H* of the prediction is equal to the cardinality of the neural network prediction, including N-BEATS. With the increase of prediction length *H*, the requirement of model computation increases rapidly and the ability of model expression decreases. Therefore, in order to effectively predict the sequence portion of the desired horizon *H*, and ease the computational burden. N-HiTS proposed a time interpolation method to solve the problem. First, redefine the dimensions of the prediction coefficient based on the expressivity ratio *r*_*l*_ of the number of parameters per unit output time, $\left|\theta_{l}^{f}\right|=\lceil r_{l}H\rceil$. Secondly, in order to be able to predict all points in the event horizon *H* and recover the initial sampling rate of the signal, the interpolation function *g* is used:

$$\left\{ \begin{array}{c} {\hat{x}}_{\tau ,l}=g(\tau ,\theta_{l}^{f}),\forall \tau \in \left\{t+1,\ldots ,t+H\right\}\\ {\tilde{x}}_{\tau ,l}=g(\tau ,\theta_{l}^{b}),\forall \tau \in \left\{t-l,\ldots ,t\right\} \end{array}\right.$$ (10)

Where *g* is a linear interpolation function.

#### 2.2.4 The structure between blocks.

The inter-block connections in N-HiTS are organized using a hierarchical double residual topology comprising two distinct branches. In the first branch, the model computes the residual by subtracting the output of the previous block from the input of the next block. In the other branch line, the predicted result of each stack is a combination of the predicted result of all the blocks in it, as follows:

$$\left\{ \begin{array}{c} {\hat{x}}_{t+1:t+H}=\sum_{l=1}^{L}{\hat{x}}_{t+1:t+H,l}\\ x_{t-L:t,l+1}=x_{t-L:t,l}-{\tilde{x}}_{t-L:t,l} \end{array}\right.$$ (11)

**Fig 7:**
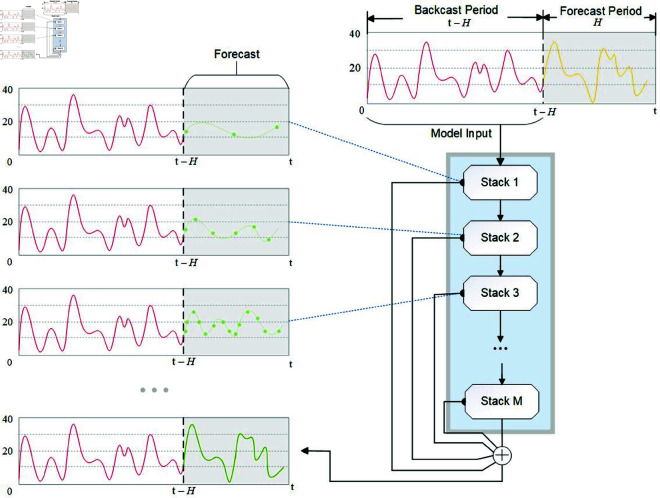
Local predictions are combined into global predictions.

Different blocks exhibit different time scales, and each block only focuses on the scale of its own input signal and output signal. Therefore, the structure of prediction results after combination is clear and the prediction effect is more accurate, as shown in Fig 7.

## 3 Results

### 3.1 Data collection

**Fig 8:**
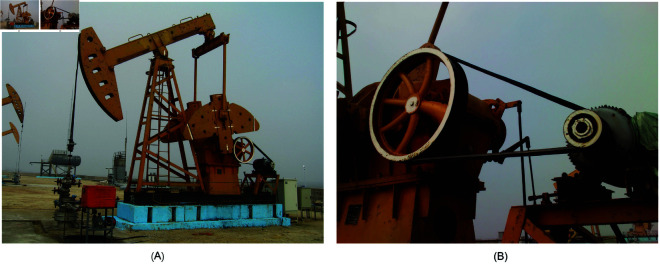
Rod pumping unit. (A) Structure of rod pumping unit. (B) Drive motor and gearbox.

**Fig 9:**
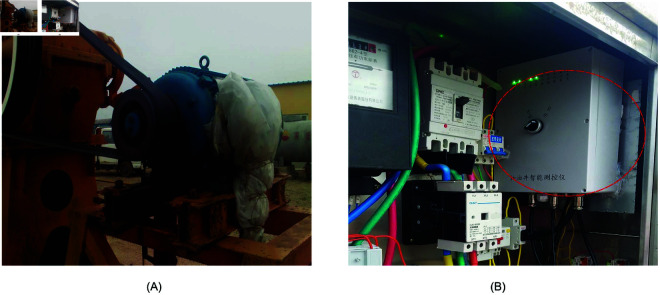
Drive motor and parameter sensor. (A) Drive motor. (B) Sensors installed in the distribution box.

The data used in the forecasting model consists of two parts: actual electrical parameters collected from industrial field operations, and generated from DCs. The operation of a beam pumping unit and the rotation of its motor need to maintain relative mechanical balance. In China, the commonly used beam pumping units primarily adopt three types of balancing methods: walking beam balance, crank balance, and compound balance. As shown in Fig 8, the crank-balanced beam pumping unit selected in this study comes from an oilfield site in China. The actual electric parameters are obtained through the sensor installed in the distribution box of the driving motor, which can collect the power, current and voltage parameters of the motor operation and perform simple data processing. The acquisition of electrical parameters is achieved through current transformers (CTs) and voltage transformers (VTs). An STM32 microcontroller serves as the embedded processing platform, enabling precise computation and analysis of key indicators such as electrical parameters and system balance. The sensors that drive the motor and collect the parameters are shown in Fig 9. In contrast to sensors installed in other parts of the pumping unit for collecting DCs, the sensor for collecting electrical parameters can be powered by a distribution box. Therefore, the electrical parameter sensor can work continuously and obtain electrical parameter data in real time. The electric parameter sensor is installed in the distribution box to effectively prevent the possibility of damage to the sensor in the harsh environment of the oil field.

Before the study of the electric parameters, the DC is the main data to study and analyze the operating state of rod pumping unit. Therefore, the existing data sets of DCs contain many types of fault conditions. In a single dynamometer card, the load change time of the suspension point is a complete stroke. The paper collects the data set of the DC recorded by an oil well, which contains several fault types, and the load variation of each DC is extracted as the input of the electric parameter generation model. The motor is a three-phase induction motor, the shaft diameter of the motor is about 28 mm, the base hole distance is 190×140 mm, and the center height is about 112 mm. The rated parameters of the motor are shown in the Table 1. The specific model of the rod pumping unit is CJY10-3-53HB.

**Table 1:** Parameters for driving the motor.

Rated power/W	Rated voltage/V	Rated speed/r · min^−1^	Rated torque/N · m
4000	400	1430	26.6331

**Fig 10:**
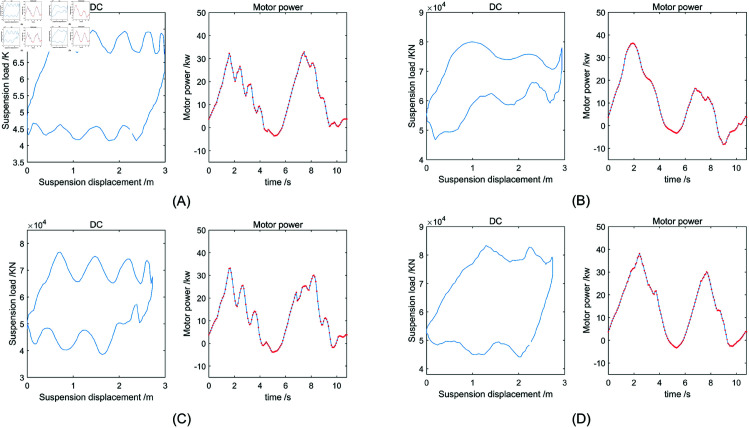
DC and converting the resulting electrical power curve. (A) Normal. (B) Insufficient fluid supply. (C) Gas influence. (D) Leakage from the pump.

In the experiment of generating motor power, the peak voltage supply of the motor is set to (400$\times \sqrt{2}$)/$\sqrt{3}$, the frequency is set to 50 Hz, the phase difference is set to $120^{\circ }$, and the initial speed is set to 1480 r/min. Examples of generated normal and faulty motor power data are shown in Fig 10. The fault conditions include insufficient liquid supply, gas influence and leakage from the pump. In addition to this type of fault condition, the electric parameter generation model can simulate the equilibrium state. The equilibrium condition is defined as the motor performing equal amounts of positive work during both the upstroke and downstroke phases. For the crank balanced rod pumping unit, a counterweight is added to the outer end of the crank shaft. In the down stroke phase, the motor drives the rod down. Since the rod has significant weight and the upward buoyant force acting on the plunger is insufficient to fully counteract it, the net downward force aids in the motion. As a result, the power demand on the drive motor is low during the downstroke. If there is no counterweight, the larger rod weight, there may be reverse work on the motor. The added counterweights rotates with the crank shaft from the six o’clock direction to the twelve o’clock direction, not only to prevent the reverse work on the motor, but also to store energy during the down stroke. During upstroke, the rod pumping unit needs to raise the rod and liquid load, so that the output power of the driving motor increases. At this point, the counterweight returns to the six o’clock direction, releasing the stored energy to help the motor lift the rod. The balance method mentioned above is very important in the normal operation of the pumping unit. In actual production, the balance degree of the pumping unit is allowed to fluctuate within a certain range. However, if the balance is damaged beyond the reasonable range, the pumping unit will likely produce more serious failures, such as severe vibration of the pumping unit and motor damage. The balance problem may occur alone, or may be accompanied by other fault types, that is, compound fault. The unbalanced state is reflected in the motor power curve to produce an obvious difference in the peak of the up and down strokes. The electric parameter generation model proposed in this paper can simulate different equilibrium states, balance fault and compound problems by adjusting the size of the balance weight.

### 3.2 Prediction of actual electrical power data

In field applications, electrical parameters are collected by sensors installed in the distribution box. During operation, the motor drives the crankshaft at low speed via a gearbox, resulting in high-frequency electrical data due to the continuous motion of the rod pumping unit. However, much of this data is repetitive and redundant, as the unit operates in a stable, periodic manner. Transmitting all raw data to the STM32 microcontroller not only consumes substantial bandwidth and storage but also poses challenges for long-term forecasting. According to the periodicity of pumping unit operation, a downsampling method to reduce the length of measured electric parameter sequence is proposed in this paper. In the operation monitoring of the pumping unit, the stroke cycle is set to approximately 6 seconds. Adopting intermittent sampling mode, that is, first pause for 30 s, then sample a complete working cycle (6 seconds), and then pause for 30 s, and repeat this cycle. According to calculations, 100 electric parameter samples can be collected per hour.

**Fig 11:**
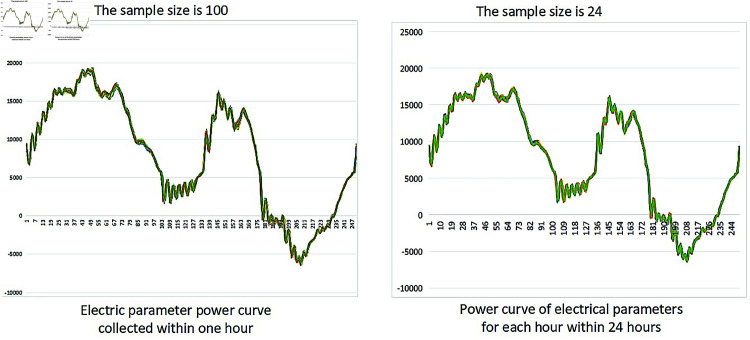
Statistical results of measured motor power data in one day.

Taking electrical power as a reference, conduct statistical analysis on electrical parameter data within a month. As shown in Fig 11, the shape of the electric power samples collected within one hour and the shape of the representative samples for each hour of the day were recorded. It can be seen that in the collected electrical parameter samples, the motor power within one hour is basically the same. On the same day, the motor power is also the same for every hour. Therefore, the electrical parameter data of one day can be replaced by the average value of the electrical parameter in a day. To prevent large amounts of redundant data from occupying the limited memory space of the STM32, the system is designed for real-time monitoring and edge processing. Each sample is analyzed in real time: if a fault is detected, relevant fault data will be immediately displayed on the screen; Otherwise, only the daily average value will be stored, not all raw data. This approach significantly improves the efficiency of both real-time monitoring and edge computing. Firstly, record the average motor power for one cycle within each hour. Then, the hourly averages are aggregated and averaged again to compute a daily representative cycle power value. N-HiTS model was used to verify the sampled electrical parameters. Due to the fact that predictive models (N-HiTS) only use data from local time windows for training and prediction, sample imbalance has limited impact on the model.

**Fig 12:**
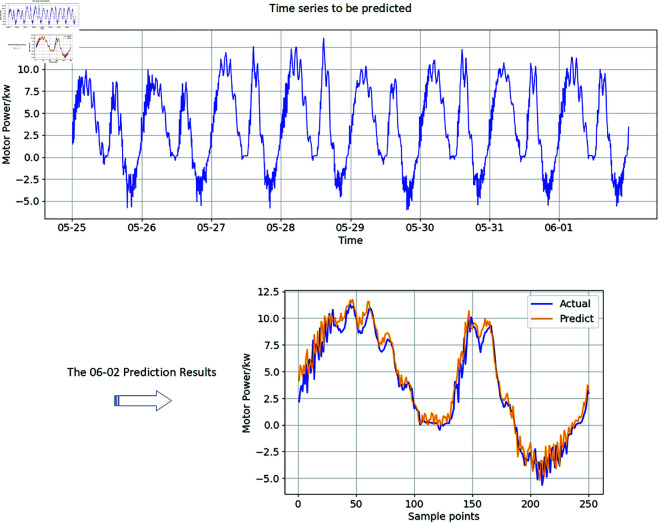
Electric power time series prediction results after sampling.

Fig 12 shows that, the predicted results of N-HiTS are close to the true values. It shows that the approach proposed in this paper can availably reduce the number of samples of electrical parameter data and reduce the calculation burden.

**Fig 13:**
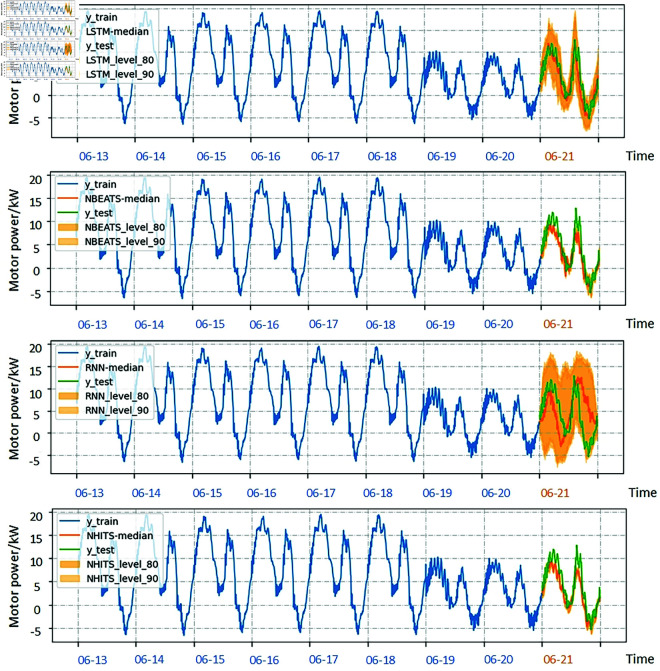
Motor power time series predicted by different forecasting methods.

Based on the above sampling methods, the real parameter data were sampled and combined into 8 time series, and the N-HiTS model was used to make probabilistic prediction of 8 time series. The prediction results included the predicted value and prediction range. Three time series prediction models, N-BEATS, RNN and LSTM, were selected for comparison. The evaluation indexes of model prediction are expressed by mean square error (MSE), mean absolute error (MAE) and root mean square error (RMSE) [23]. In addition, the relative mean absolute error (RMAE) [24] is used as a comparison between the prediction accuracy of N-HiTS and other models, with a value of RMAE less than 1 indicating that the prediction accuracy of the selected model is less than that of the N-HiTS model. The definition of RMAE is as follows:

$$RMAE(y_{\tau },{\hat{y}}_{\tau },{\hat{y}}_{\tau }^{base})=\frac{1}{H}\sum_{\tau =t+1}^{t+H}\frac{\left|y_{\tau }-{\hat{y}}_{\tau }\right|}{MAE(y_{\tau },{\hat{y}}_{\tau }^{base})}$$ (12)

where $y_{\tau }$ is the observed values; ${\hat{y}}_{\tau }$ is the predicted values of first model; ${\hat{y}}_{\tau }^{base}$ is the predicted values of baseline model;and the *H* is the length of predicted results. Each model is trained eight times, the number of iterations per training is 1000, and the prediction length is one period. Choose the one with the best prediction effect to record. The effect of prediction shows in Fig 13.

In Table 2, the evaluation indicators of the predicted results are listed. It can be seen that the accuracy of N-HiTS prediction results is higher than the other three models.

**Table 2:** Evaluation of prediction results of actual motor power parameters.

Model	MAE	MSE	RMSE	RMAE
LSTM	1.139	2.387	1.545	0.785
NBEATS	0.906	1.599	1.264	0.974
RNN	4.681	32.576	5.708	0.193
N-HiTS	0.833	1.501	1.225	1

The measured electrical parameters are typically continuous and evolve slowly over time. Consequently, accurate forecasting relies on the accumulation of long-term historical data to effectively capture gradual trends and subtle variations. Given this temporal nature, it becomes essential to generate and incorporate representative fault-related samples into the training process. This ensures the model is exposed to abnormal conditions that may not frequently occur in the original dataset, thereby enhancing its ability to generalize and detect fault signatures during inference.

### 3.3 Generate parameter prediction

**Fig 14:**
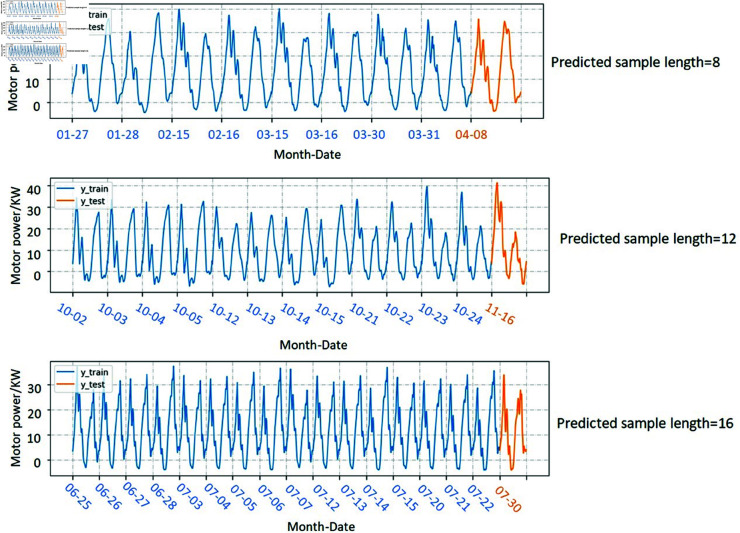
A combination of three lengths of motor power time series.

Combine the electrical parameter data generated by DC into a time series and use it for predicting experiment. Eight time series, each consisting of four categories of motor power data, are constructed as training samples. To evaluate the model’s forecasting performance across different time series lengths, three training sets with varying durations were created. The way to change the length of the time series is to modify the number of days for each condition in the time series. In the first set, each time series includes four categories of motor power data, with two days of data per category. The second set of time series adds one day to each type of motor power data on the basis of the first set of time series. In the third group, the parameters of each type of motor are extended to 4 days. For each group, a one-cycle motor power time series is predicted, as illustrated in Fig 14. In the electric power sequences in combination, the X-axis represents the time when the DC is collected.

**Fig 15:**
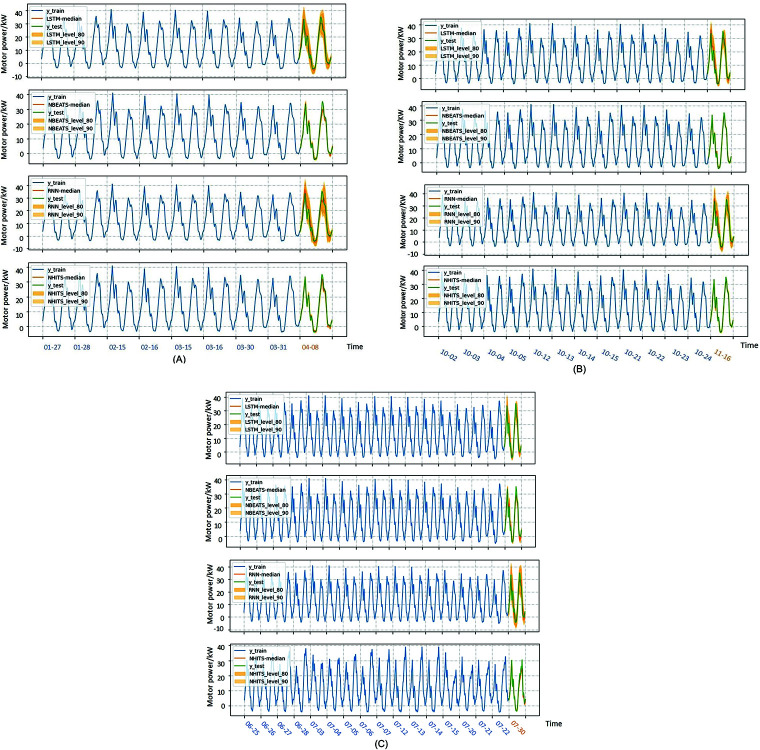
Experimental results with a predicted length of one period. (A) The motor power sequence length is 8 days. (B) The motor power sequence length is 12 days. (C) The motor power sequence length is 16 days.

**Fig 16:**
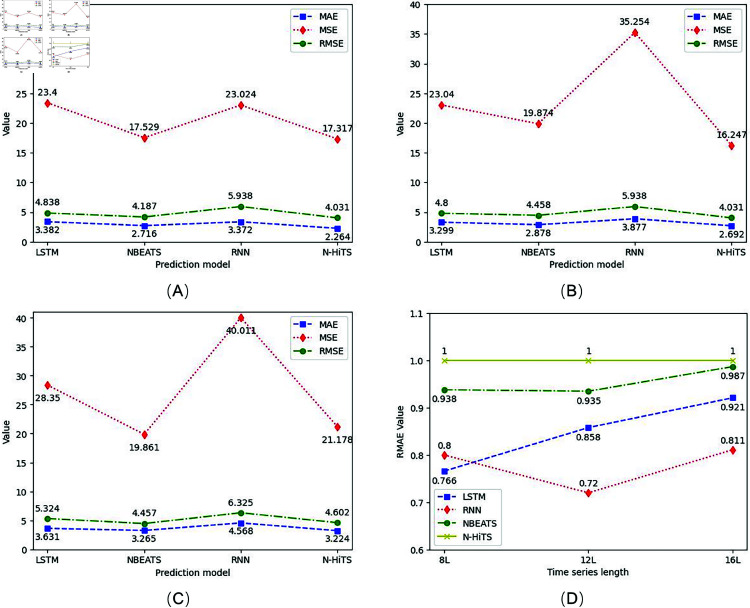
Comparison of model prediction results. (A) An 8-day evaluation index of prediction results. (B) An 12-day evaluation index of prediction results. (C) An 16-day evaluation index of prediction results. (D) The N-HiTS model compared with the predictions of other models.

So as to verify the feasibility of generating electric parameter combination as motor power, the time series of the combination was first predicted in a single period, and the prediction results were shown in Fig 15. The evaluation indicators of the predicted results were shown as Fig 16.

From the prediction results and evaluation indexes, it can be concluded that the prediction accuracy of N-HiTS model is better than the other three prediction models. N-HiTS model can not only predict the time series of motor power more accurately, but also has the characteristics of fast operation speed. As shown in Table 3, the running time of a three-length time series is measured in seconds. Under the same experimental conditions and prediction targets, the prediction speed of N-HiTS model is significantly lower than that of LSTM and RNN, and better than N-BEATS.

**Table 3:** A three-length sequence of parameters is used to predict the time spent in seconds.

Model	8 periods	12 periods	16 periods
LSTM	115.38	115.06	92.18
RNN	77.21	104.08	150.46
NBEATS	38.60	39.99	42.42
N-HiTS	37.38	38.03	39.62

**Fig 17:**
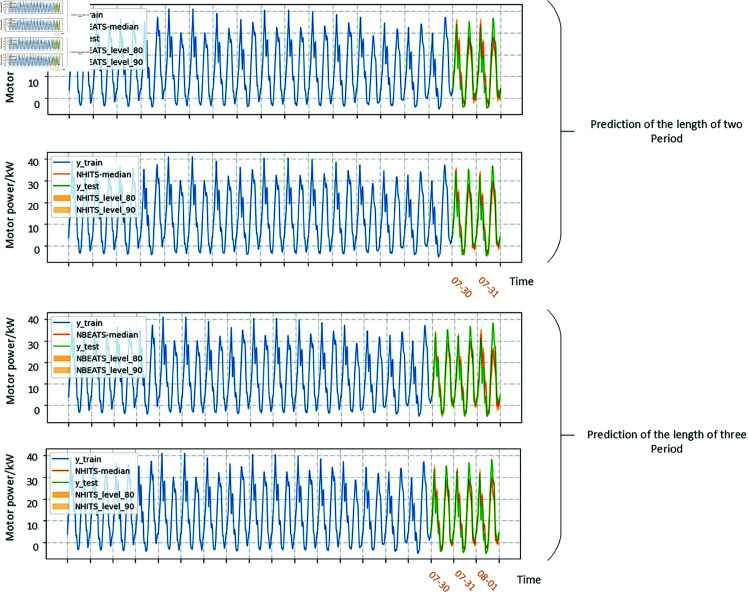
Multi-period forecast results.

**Fig 18:**
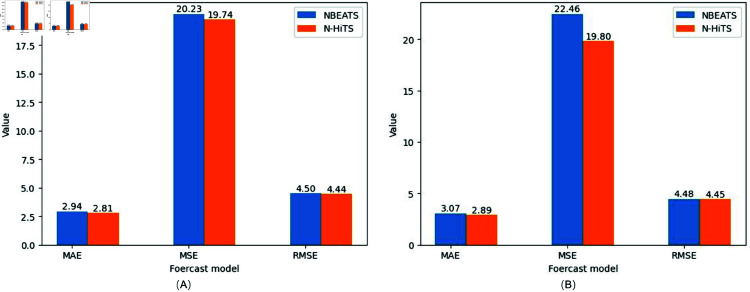
Evaluation of Multi-period prediction results. (A) An evaluation index with a prediction length of 2 days. (B) An evaluation index with a prediction length of 3 days.

For identifying the prediction of multi-period, the generated parameter time series of the third combination is used as input to predict the electrical power data for the next two and three days, as shown in Fig 17. The indicators for the predicted results are shown in Fig 18.

When the length of the forecast is three periods, the computing requirements of RNN and LSTM increase rapidly, and the burden of the hardware system is too large to meet the purpose of the forecast. Therefore, the prediction results of RNN and LSTM for multiple periods are omitted, because they consume significantly more time and resources than the N-HiTS model under the same hardware conditions. N-BEATS compared to N-HiTS, RMAE was 0.954 and 0.942, respectively, in the results of the two multi-period predictions. N-HiTS hierarchical decomposition and multi-resolution design allow it to effectively capture both short-term fluctuations and long-term dependencies, which are often under-modeled by recurrent approaches. Compared to N-BEATS, which also adopts a block-based forecasting strategy, N-HiTS introduces interpolation-based skip connections and multi-scale aggregation, leading to improved accuracy and better generalization. While N-BEATS tends to smooth over high-frequency patterns, N-HiTS preserves these features through its refined bottom-up hierarchical learning scheme. Therefore, N-HiTS provides a superior architecture that balances interpretability, forecasting accuracy, and computational efficiency, making it well-suited for high-resolution and fault-sensitive industrial time series applications.

### 3.4 Fault state prediction

Compared with the measured electrical parameters, the generated electrical parameters contain more fault conditions. In reality, the pumping unit consists of underground equipment and surface equipment, and the surface equipment includes rod pumping units and driving motors. Therefore, the fault state reflected by the pumping unit may be a combination of multiple fault types. The electric parameter generation model proposed in this paper can generate complex conditions with balance problems and downhole faults. For the balance problem, the motor power curve can better reflect whether the pumping unit has overbalance and underbalance problems. The mechanical balance of the pumping unit is primarily achieved through crank counterweights. These counterweights are attached to the crank arm and serve to offset the dynamic load exerted by the reciprocating motion of the polished rod and sucker rod string. By adjusting the mass and angular position of the counterweights, the torque fluctuations experienced during operation are significantly reduced, thereby improving energy efficiency and ensuring smoother rotational motion of the gearbox.

**Fig 19:**
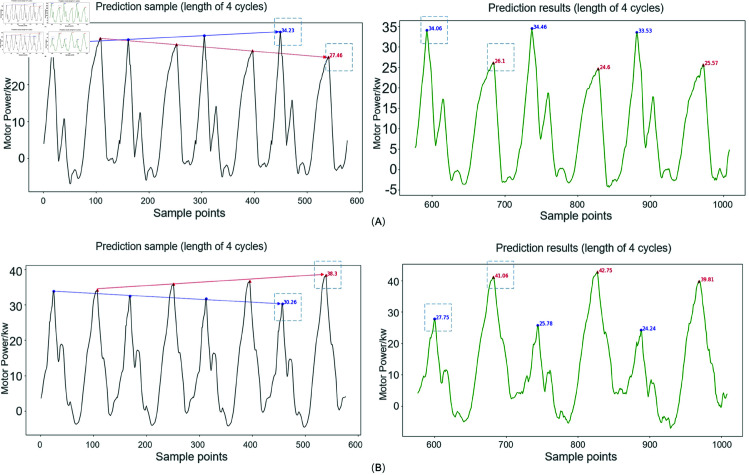
Prediction of composite fault states. (A) Prediction of the composite state of gas influence and overbalance. (B) Prediction of the composite state of traveling valve leakage and underbalanced.

In most cases, changes in the environment, underground working conditions, and the machinery itself can affect the state of balance. So, the pumping unit cannot maintain an ideal equilibrium state for a long time. Similarly, it is also not advisable to frequently adjust the balance state to meet ideal conditions. Therefore, based on long-term experience and the physical significance of electric power, it is set that in the electric power curve, if the ratio of the smaller peak to the larger peak is greater than or equal to the balance threshold of 0.8, the balance state of the pumping unit is judged to be good and does not need to be adjusted. From this, it can be concluded that the definition of the overbalance state is that the peak power of the motor during the upper stroke is greater than the peak power during the lower stroke. And the ratio of the peak power during the lower stroke to the peak power during the upper stroke is significantly less than 0.8. Underbalance, on the other hand, occurs when the peak value of the upper stroke is smaller than that of the lower stroke, and the ratio of the peak value of the upper stroke to the peak value of the lower stroke is significantly less than 0.8. The severity of the fault can be judged by predicting the generated compound working conditions. In addition, the causes of existing fault states can be analyzed to determine the composition of compound conditions. For example, this paper generates motor power data for two compound fault types: Gas influence and overbalance; Traveling valve leakage and underbalanced. Set a gradually changing equilibrium state for the fault power and merge it into a time series. Predict the electric power data for the next three cycle lengths. Two trends of increasing severity of overbalance and underbalance were identified separately. The predicted results are shown in Fig 19.

**Fig 20:**
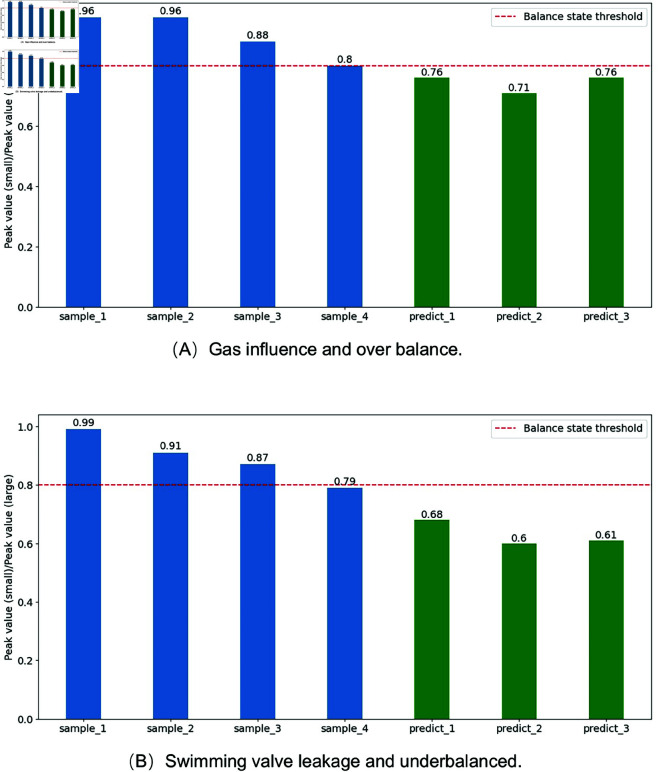
The ratio of smaller peak to larger peak in the prediction experiment.

The ratio of the smaller peak to the larger peak for the two composite operating conditions is shown in Fig 20. The degree of change in the two types of composite fault conditions varies. As shown in Fig 19B, while the traveling valve is leaking, the predicted unbalanced fault situation is significantly lower than the threshold of 0.8, and the imbalance situation gradually becomes more severe. Gas influence may alter the suspension load by introducing compressibility and phase disturbance effects during the pumping cycle. This can exacerbate mechanical imbalance, especially when the crank counterweight settings no longer correspond to the real-time dynamic loading. If left uncorrected, such imbalance accelerates wear on the mechanical system and increases the risk of early-stage failure in critical components. The fault changes in Fig 19A, although the predicted results are also less than the threshold of 0.8, have a relatively mild degree of imbalance. In practical applications, the electromechanical parameter generation model can simulate the process of gradual imbalance. Combining predictive models can help technicians identify critical fault nodes, schedule maintenance schedules, and save time and economic costs.

DC has a long history and contains many fault samples of rod pumping units. By converting the mechanism model into electrical parameters, the problem of insufficient electrical parameter samples under fault conditions has been solved. The electric parameter generation model can simulate the balance state, and can generate the compound fault condition under the existing fault state. Compared with the DC, the fault types of the generated electrical parameter data can be more diversified. In addition to the combination of the generated parameter sequence, the actual motor parameters are reasonably sampled. Through the comparison experiment, it is found that the periodicity of the parameter has little influence on the prediction result. Therefore, it is an effective solution to solve the problem of excessive data of measured electrical parameters to combine and predict the extracted actual electrical parameters. Finally, the N-HiTs model is used to predict the generated fault parameters and sampled the actual parameters. So as to identifying the predictive ability of the model, the length of the predictive time series is set differently and compared with the typical predictive model. From the experimental results, N-HiTS model has good predictive ability. In the actual sequence of electric parameters, N-HiTS still showed accurate prediction effect. In addition, N-HiTS is faster and requires less resource consumption than other predictive models. N-HiTS provides an accurate and fast deep learning method for predicting the electric parameters of rod pumping unit motors.

## 4 Conclusions

This paper presents a novel method to predict the electric parameters of rod pumping unit. This paper uses the mechanism model to generate the electric parameters of rod pumping unit and combines them into a time series. The measured electrical parameters are reasonably sampled. The deep neural network model N-HiTs was used to predict the time series.

Compared with DC, the electric parameters can be obtained easily, with low cost, and can be collected stably for a long time. By fault prediction of the generated motor parameters, the degree of fault variation can be observed. The prediction of the compound fault state can help technicians to analyze the cause of the fault, so as to carry out reasonable maintenance. The rod pumping unit needs continuous operation to ensure the production target, and the electrical parameter data are long and huge for the fixed electrical parameter acquisition equipment. The sampling method proposed in this paper can availably reduce the amount of data and realize the prediction of actual parameters. However, several limitations must be acknowledged. Over extended forecast horizons, such accumulated errors could lead to deviations. Additionally, the model has not yet been validated under abrupt mechanical fault conditions, and its performance in such extreme scenarios remains uncertain. In the future research, we can classify the predicted results of the motor parameters of the pump group, and then judge the potential failure problems of the pump group, and arrange production maintenance in a planned way to reduce economic losses and improve production efficiency.

## Supporting information

S1 Data Dynamometer card and and electrical parameter data. The data comprises dynamometer card sensor measurements and electrical parameters monitored by oil well production sensors.
(ZIP)
